# Validity of MRI and Ultrasound Volume Measurements of Foot Muscles and Plantar Fascia Cross‐Sectional Area Within Older Adults With and Without Chronic Plantar Fasciitis

**DOI:** 10.1002/jfa2.70076

**Published:** 2025-09-10

**Authors:** Derek A. Swanson, Joshua K. Sponbeck, Dallin C. Swanson, Steven P. Allen, Aaron Wayne Johnson

**Affiliations:** ^1^ Department of Exercise Sciences Brigham Young University Provo Utah USA; ^2^ Electrical and Computer Engineering Ira A. Fulton College of Engineering Brigham Young University Provo Utah USA

**Keywords:** chronic plantar fasciitis, intrinsic foot muscles, ultrasound, validity, volume

## Abstract

**Introduction:**

Intrinsic foot muscles and the plantar fascia are crucial for foot health, which diminishes with age and conditions such as chronic plantar fasciitis (PF). Ultrasound (US) is an accessible and cost‐effective method for evaluating these structures. This study aims to assess the repeatability, reliability, and validity of plantar fascia thickness and flexor digitorum brevis (FDB) muscle measurements using US compared with MRI in individuals with and without PF.

**Methods:**

Foot muscle volume and plantar fascia thickness were measured via US and MRI in 28 participants with and without PF. Subsequently, the plantar fascia thickness and FDB volume were calculated using the OsiriX semiauto volume segmenter software for MRI and the truncated cone formula for both MRI and US. Intraclass correlation coefficients (ICCs), Pearson product correlations (*r*), minimal detectable differences (MDD), and standard error of measurement (SEm) were calculated.

**Results:**

High ICCs (*r* = 0.988–0.990) indicated excellent repeatability for all measurement techniques of the plantar fascia and FDB muscle. Reliability for plantar fascia and FDB measurements ranged from 3.98% to 5.50% and 5.06%–9.84%, respectively, across both groups. Validity was high with correlation values between 0.94 and 0.99 and Bland–Altman limits of agreement ranging from 2.6% to 9.2%.

**Conclusions:**

US provides repeatable, reliable, and valid measurements of plantar fascia thickness and FDB muscle volume compared with MRI. It offers a cost‐effective and accessible alternative for assessing foot health in clinical and research settings.

## 1 Introduction

The intrinsic foot muscles and plantar fascia are key components of overall foot health and movement capability [1]. Intrinsic foot muscles are used to maintain balance [2], stabilize the foot [3], and support the structural integrity and arch of the foot [4]. The plantar fascia supports the arch of the foot and assists in maintaining normal foot mechanics [5]. Weakness in the intrinsic foot muscles and pathology of the plantar fascia are associated with biomechanical problems in the foot and leg, some of which include reduced shock absorption, altered gait stance [6, 7], poor balance, diabetic neuropathy [8], decreased foot muscle volume [9], foot drop, and genu recurvatum [10]. Intrinsic foot muscle weakness and plantar fascia pathology become more prevalent with age and contribute to reduced mobility [11, 12], decreased independence [12], and diminished sense of well‐being and quality of life [11].

Obtaining valid, accurate, and reliable measures of plantar fascia thickness and intrinsic foot muscle strength are vital components in understanding overall foot health as individuals age [13]. Obtaining plantar fascia thickness measurements using MRI or US has been established in the body of literature although a validity comparison of the US measures to MRI has not been reported. Direct measurement of intrinsic foot muscle strength presents a unique challenge due to the difficulty of isolating the individual muscles [14]. In addition many intrinsic foot muscles share common osteokinematic actions with extrinsic foot muscles making it difficult to differentiate individual muscle contributions during movements [15, 16]. Therefore, indirect measures of muscle strength and function, such as muscle cross‐sectional area (CSA), thickness, or muscle volume, become important tools to understand intrinsic foot muscle strength and function. Historically, studies [15, 16, 17] have employed indirect measures of muscle CSA or thickness to understand intrinsic foot muscle strength properties in healthy young adult populations.

Muscle volume is a stronger predictor of muscle strength and function than muscle CSA [18] and can be obtained utilizing MR imaging and US imaging. Volume of the FDB muscle has been calculated previously using US and a truncated cone formula and was deemed to be a reliable method for calculating volume [19]. Volume of the FDB has also been calculated by using MR imaging and a truncated cone formula [20, 21] while comparing healthy runners and runners demonstrating chronic plantar fasciitis [9]. As of current US and MR imaging modalities regarding the FDB muscle volume and plantar fascia thickness have not been directly compared to one another in either healthy or pathologic groups. Establishing the validity, reliability, and repeatability of US measures of intrinsic foot muscle volume and plantar fascia thickness to MRI in healthy individuals and those with plantar fasciitis is vital to aid clinicians and researchers that use US imaging during assessment and rehabilitation. This need only grows as US continues to gain clinical popularity due to its ease of use and availability compared with MRI.

There are various methods to estimate muscle volume using a smaller number of images each with varying accuracy and precision. Two methods, the truncated cone and cylinder or Cavalieri, have been used to measure volume in other muscles of the body [9, 22, 23, 24, 25]. Researchers have assessed muscle thickness [14], CSA [26], and combined forefoot and rear foot muscle volume [27], showing high levels of reliability [20, 28, 29] and validity of cross‐sectional area measurements compared to MRI [30]. Although these measurements have been shown to be reliable and useful clinically and academically, they are still limited to one or two dimensions.

Volume and shape of a muscle are strongly related to its function [31, 32]. Muscle volume is also recommended for studying strength training rather than using CSA [33]. Muscle size increases have been positively correlated with muscle strength and function [17, 34, 35]. However, calculating muscle volume, can be a time‐consuming process if needing to individually measure every image and may not be clinically feasible due to time or equipment constraints. For example, one study examined quadriceps muscle volume using MRI and reported 370 h of analysis required to run three replicates for both legs of 42 subjects [36]. Besides time constraints many clinicians do not have direct access to an MRI nor the software to analyze MRI files.

Using a formula to combine multiple muscle CSA's and determining a complete muscle volume could lead to an accurate and reliable estimation of volume and subsequent strength within individual muscles while decreasing time spent analyzing. These prior studies mentioned were conducted using a healthy young population as their participants; however, many pathologies are more commonly found in older populations. Thus, assessing reliability and validity of intrinsic foot muscle volume measurements is important in an older population [37].

The plantar fascia assists in supporting the arch of the foot and plays an important role in foot mechanics and gait [38]. Asymptomatic or regular plantar fascia thickness varies typically between 2.2 and 4.0 mm [39, 40] whereas fascia thickness of over 4.0 mm [41] is commonly symptomatic for plantar fasciitis or a more general plantar fasciopathy [42]. Different thicknesses of the plantar fascia have been used to determine pathology with this pathological thickness being greater than 5.0 mm [42, 43]. Plantar fasciopathy is common in the older adult population [44] and can occur due to a variety of factors, some of which include a sedentary lifestyle and obesity [45, 46]. Persons with plantar fasciitis tend to have diminished muscle volume [9]. Thus, there seems to be a relationship between plantar fascia structure and muscle morphology and function in the foot in some pathological conditions.

Therefore, the objective of this study is to assess the repeatability, reliability, and validity of (1) plantar fascia thickness and (2) of a truncated cone volume formula during measurement of an intrinsic foot muscle, the FDB, and between US and MR imaging in individuals with and without PF. Given MRI's established role as a reference standard for muscle volume evaluation, it will serve as the criterion measure. We hypothesize that (1) measurements of plantar fascia thickness between US and MRI will show a very strong correlation suggestive of high validity, along with excellent repeatability and reliability. We hypothesize (2) that US based measures of intrinsic foot muscle volume utilizing a truncated cone formula will show a very strong positive correlation with MRI based measurements obtained through both manually calculated and semiautomated (OsiriX) truncated cone formula in both groups. We expect repeated volume measurements from both US and MRI to demonstrate an excellent level of reliability and repeatability.

## 2 Materials and Methods

Our study is a prospective cross‐sectional validation study evaluating the repeatability, reliability, and validity of ultrasound measurements of plantar fascia thickness and flexor digitorum brevis muscle volume compared with MRI. Analysis included ICCs, Pearson correlation coefficients, and Bland–Altman plots to assess agreement and consistency between modalities. Plantar fascia thickness and FDB volume were measured via US and MRI in 28 participants (females = 16 and males = 12) (age 54.6 ± 6.63). Thirteen of these participants had at least one foot diagnosed with PF (females = 6 and males = 7) (age = 52.08 ± 5.53) and 15 individuals without PF (females = 10 and males = 5) (age = 54.70 ± 6.79). In total, 18 feet were included in the PF group and 38 were included in the non‐PF group. A total of 30 participants were originally recruited for the study, two participants were excluded due to study protocol adherence. Participants were recruited through campus‐wide advertisements. We had no prior contact with any individuals before their expression of interest. Those who believed that they met this study criteria voluntarily reached out to the research team to participate. This study inclusion criteria were as follows: at least 45 years of age, with healthy feet (no lower extremity injury within the last 1 month or leg/foot surgery within the last 3 months) or with PF (PF of a participant was diagnosed by a United States board‐certified or board‐qualified physician beforehand), able to be imaged by an MRI scanner and not pregnant. Any study participant was excluded who reported lower extremity injury within the last 1 month or leg/foot surgery within the last 3 months.

Each participant read and signed an informed consent approved by the University's Institutional Review Board (study protocol, IRB#2022‐332). All imaging was completed in one session consisting of two parts. MR imaging was conducted first. Immediately following the MR imaging session, participants completed the US imaging portion. Both sessions were completed within 1 hour of each other.

### 2.1 Preparation for Imaging Sessions

**FIGURE 1:**
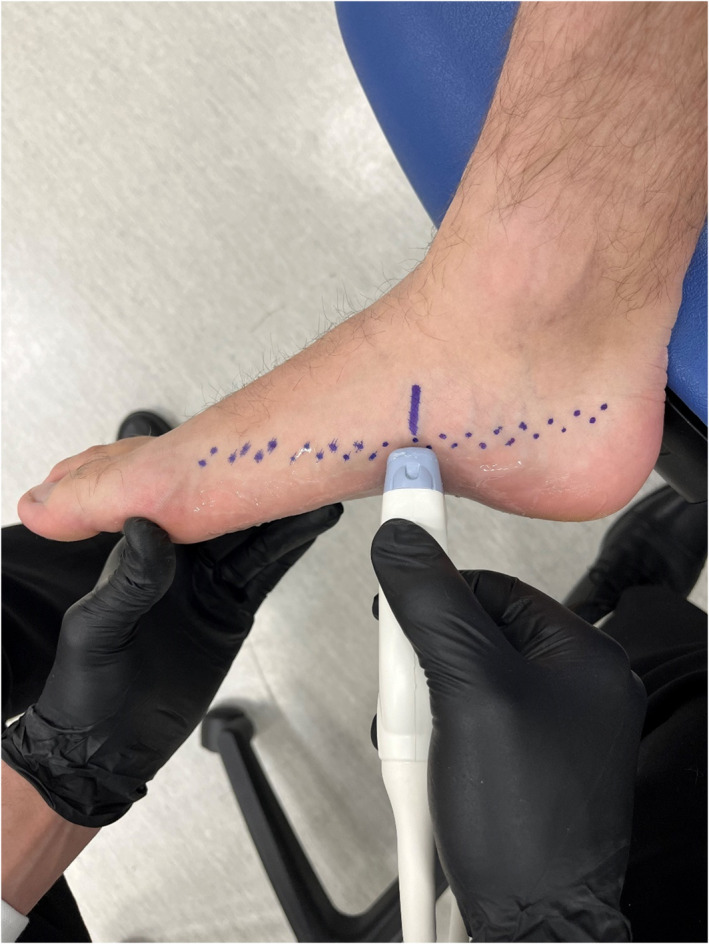
Transducer probe placement with guidance line on navicular tuberosity marking. Markings made every 0.5 cm.

**FIGURE 2:**
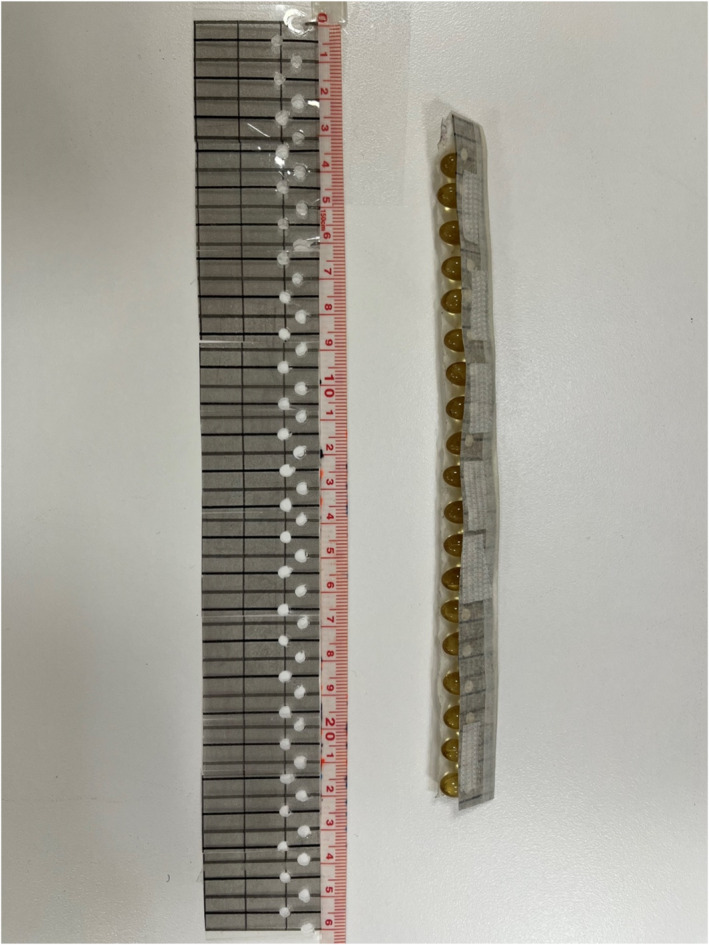
Gel capsule strip used for MR imaging and marker template for marking feet for imaging.

The reference point for US and MRI measurement locations on the foot were determined by creating a singular mark at the most prominent part of the navicular tuberosity. Based on this reference point, a stencil with slits every ½ cm was placed directly on the medial surface of the foot biased toward the plantar surface. Additional marks based on the reference point were placed along this stencil every ½ cm along the medial border of the foot in both the proximal and distal directions (Figure 1). These markings were used for US imaging and guided the placement of a fitted gel capsule strip that was utilized for MR imaging (Figure 2). The stencil was used to reduce possible marking errors on each foot of every subject.

### 2.2 US Protocol

All images taken were done by the same researcher (Author D. C. Swanson previous demonstrated Intraclass correlation coefficient (ICC) reliability for intrinsic muscle CSA range from previous publication: 0.994–0.999) who has over 3 years of US scanning experience specializing in intrinsic foot muscle imaging. An ML6‐15‐D matrix linear transducer probe (LOGIQ FORDIS; GE Healthcare, Chicago, IL) was used to take all images. US settings were kept constant with a scanning depth of 3 cm and frequency of 8 MHz. Focal position and time‐gain‐compensation were also kept constant while gain was adjusted, as needed, to improve clarity of the image. Each participant was seated in a semiupright position with a pillow placed beneath the thigh of the leg that was being imaged. The hip of the leg being scanned was abducted and the foot laid off the table at ninety‐degrees in a frog leg position. This allowed for consistent positioning of participants in imaging the medial/plantar regions of the foot.

During imaging of the plantar fascia, the ultrasound probe was placed on the plantar surface of the foot and adjusted to maximize image quality. The probe was oriented in a sagittal plane in line with the calcaneus toward the toes in long axis view. A still image was taken on each foot for measuring with the calcaneus bone in view. The thickest observed band of the plantar fascia was imaged and recorded for plantar fascia thickness.

The probe was positioned in short axis on the plantar surface of the foot. Beginning at the calcaneus, a short video recording the change in CSA and shape of the FDB muscle as the probe was scanned from the proximal attachment to the distal attachment. This captured video was referenced if needed during the data analysis to help visualize progressive changes in muscle size for subsequent slices. The FDB was imaged every ½ cm over the length of the muscle to capture individual muscle slice CSA's that were later used for estimating muscle volume. For each image taken, the transducer probe was placed in the coronal direction on the plantar midfoot surface of the foot in line with the corresponding one‐half cm marks (Figure 1). Based on the length of the participants' feet, 15–25 still images were recorded and used for analysis. These images were taken separately after the proximal to distal video was taken.

### 2.3 MRI Protocol

Each subject's feet were imaged one at a time using a single 8‐channel dedicated foot/ankle coil (Knee‐Foot Array, ScanMed, Omaha, Nebraska) with a Tim Trio Connector (Siemens, Erlangen, Germany) and inside a 3 T MRI scanner (VIDA, Siemens, Erlangen, Germany). Images of intrinsic foot muscles were captured using a T1‐weighted 3‐dimensional spoiled gradient echo sequence prescribed with the through‐plane direction perpendicular to the long axis of the entire foot. Water band excitation was used to eliminate fat signal and enhance the appearance of muscle borders. Slice thickness was 0.5 mm and slices were contiguous with no gap or overlap between them. Scan parameters were as follows: TE/TR = 10.0/4.92 ms; matrix size: 480 × 296 × 220 pixels; resolution: 0.52 × 0.50 × 0.51 mm; field of view: 240 × 154 × 110 mm; flip angle: 9°; no acceleration; bandwidth: 270 Hz/pixel; and total acquisition time: 10 min and 11 s per foot.

**FIGURE 3:**
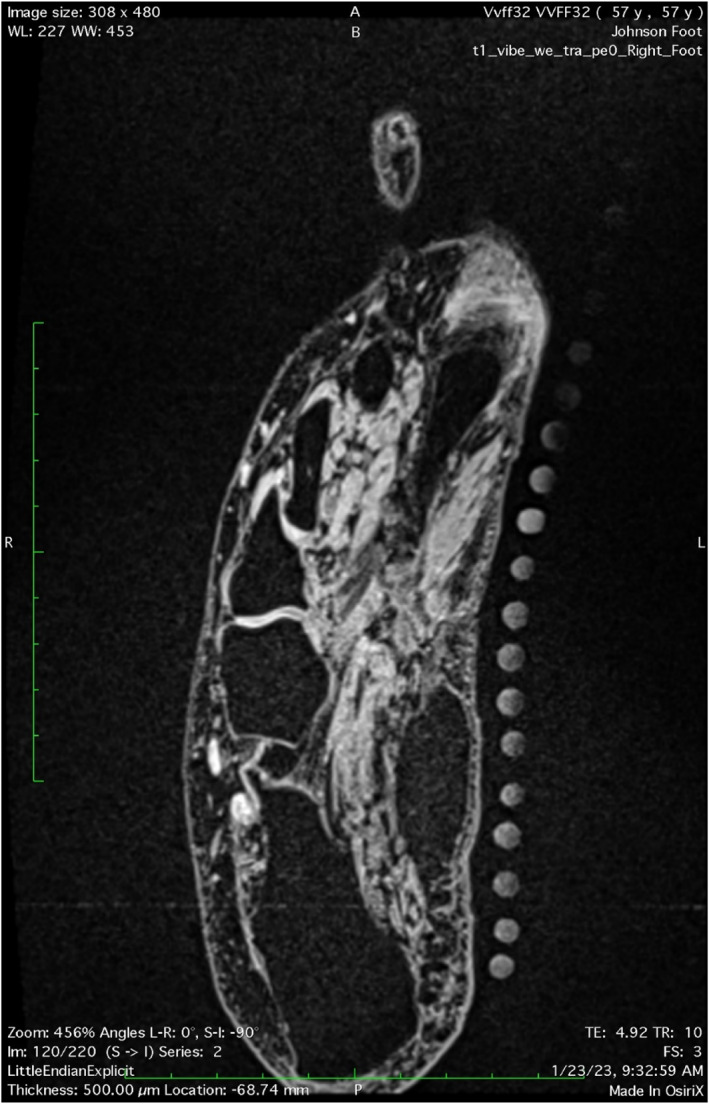
Fitted gel capsule strip on MRI.

Upon arriving, participants completed an MRI safety screening form along with other study related forms before moving forward with the scan and entering the magnet room. After the MRI safety screening form was verified and approved the markings were made on the foot using the stencil. The fitted row of 125 mcg vitamin D3 gel capsules was then positioned on the foot to ensure the reliability of imaging measurements. It was attached to the participant's skin on the inferior medial foot via double‐sided Velcro. These capsules served as markers that are visible via MRI (Figure 3). The capsules were positioned on the markings made previously. Sandbags were used to immobilize the foot during imaging sessions. Total imaging time per participant was about 30 min for both feet. The total number of slices per foot was 480 in the coronal plane. The images measured per foot varied between 15 and 25 corresponding with gel capsules and visualized FDB muscle. Images were measured for volume using the OsiriX semiauto volume segmenter software (OsiriX MD v.10.0.1, Pixmeo, Geneva, Switzerland) and the truncated cone formula.

### 2.4 Data Processing

All plantar fascia measurements were taken from two individual images. The plantar fascia thickness was calculated by measuring at the distal end of the calcaneus with a perpendicular line extending from the deep to superficial borders of the imaged plantar fascia. Measurements were taken on both images and an average of the two measurements was used to determine the plantar fascia thickness. This was done on each subject in both the control and PF group bilaterally.

MRI images were segmented every ½ cm from FDB origin to insertion and recorded. All muscle CSAs were segmented on the inside of the muscle fascia border for both MRI and US. US CSA images were segmented or measured using the internal software of the LOGIQ S8 US machine. Both MRI and US CSA's were recorded in centimeters squared. These CSAs were used to create a total volume via the MRI OsiriX semiauto volume segmenter or truncated cone formula. The total number of CSA images taken per foot ranged from 15 to 25 (Average 20.4 slices) measurements depending on muscle length.

**FIGURE 4:**
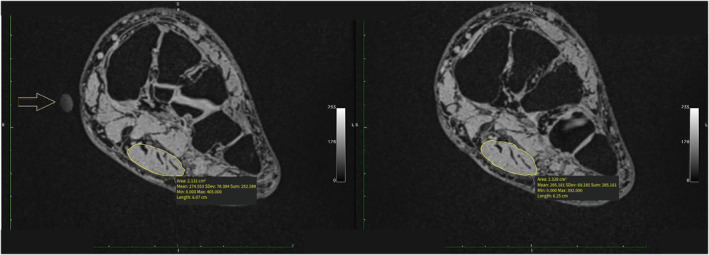
MR image depicting a slice showing gel capsule at maximum size (left) and image depicting no capsule (right).

CSA measurements of the MRI were taken at the middle of the gel capsule (the largest part of the capsule on the slice images), and the middle in between two gel capsule areas, where no gel capsule could be seen (Figure 4). Measuring at both these locations corresponded to US imaged CSA slices. Measurements were repeated twice for all five imaging groups: MRI OsiriX semiauto volume segmenter (MRI OsiriX), MRI truncated cone, ultrasound truncated cone, plantar fascia MRI, and plantar fascia US. All MRI measurements were done by the same author (Author D. A. Swanson).

A muscle volume was calculated using the truncated cone formula,

(1) $$V=\left(1/3\right)\;*\;\pi \;*\;h\;*\;\left(r^{2}+r\;*\;R+R^{2}\right).$$

This same formula has been used to measure volume of various other muscles in the body [22, 36]. Where *R* is the radius of the base of the original cone (bottom surface); *r* is the radius of the top surface; and *h* is the height of our truncated cone. This means that the *R* was the radius of the previous segment, and r was the radius of the newly measured segment. This allows for a volume to be calculated quickly without compromising accuracy (reference). This formula was used for both MRI and US images where a truncated cone volume was calculated and were labeled “MRI truncated cone” and “US truncated cone,” respectively.

The OsiriX semiauto volume segmenter uses the cylinder or Cavalieri formula, V = Bh. Where V is the volume of the muscle, B is the area of a cross‐section, and h is the height. This formula assumes the muscle is an ideal cylinder and calculates volume by multiplying surface and slice thickness and then adding up individual slice frames. This was used for MRI images and is labeled “MRI Osirix”.

### 2.5 Statistical Analysis

ICC's were calculated for MRI and US plantar fascia imaging methods, both MRI volume calculation methods (MRI OsiriX and MRI truncated cone), and the US truncated cone method. The ICC calculation model featured random subjects and fixed raters (model 3,1). Standard error of the measurement (SEm) was calculated for MRI and US plantar fascia thickness, both MRI volume calculation methods, and the US truncated cone method to identify potential error inherent in the imaging. A 95% confidence interval was calculated for ICC's and SEm. The minimal detectable difference (MDD) for all volumetric imaging and both MRI and US plantar fascia thickness was calculated. Additionally, MDD was presented as a percent to better demonstrate in relatable terms. To calculate these measurements, the following equations were utilized:

(2) $$\text{SEm}=\text{SD}\;*\;\left(\text{Sq}\;\text{rt}\;1\;–\;r_{\text{icc}}\right),$$

(3) 95%CISEm=musclemean±(1.96∗SEm),

(4) $$\text{MDD}=\text{SEm}\;*\;1.96\;*\;\sqrt{2}.$$

Pearson product correlations were run to determine the correlation between the imaging modalities. Correlations were run for the MRI and US measurements of the plantar fascia and the FDB muscle for both the PF and non‐PF groups. Bland–Altman plots were created to highlight the volume difference and mean volume between MRI Osirix, MRI truncated cone, and US truncated cone (Figure 5). Bland–Altman plots allow for visualization of any systematic errors within an imaging method, statistical differences between imaging methods, and imaging method trends. The *X* axis represents the mean volume. The *Y* axis represents the volume difference or average volume of the measurements taken from each specific imaging method (mean volume = [imaging method volume 1 + imaging method volume 2] ÷ 2). We calculated the percent muscle size via limits of agreement, which was utilized to help understand the numerical differences within the volumes determined from each imaging method. All statistical analysis was performed via the SPSS version 27.0 statistical software (IBM Corporation, Armonk, NY). An alpha of 0.05 was employed to determine statistical significance.

FIGURE 5Bland–Altman plots and correlation graphs.

**Figure:**
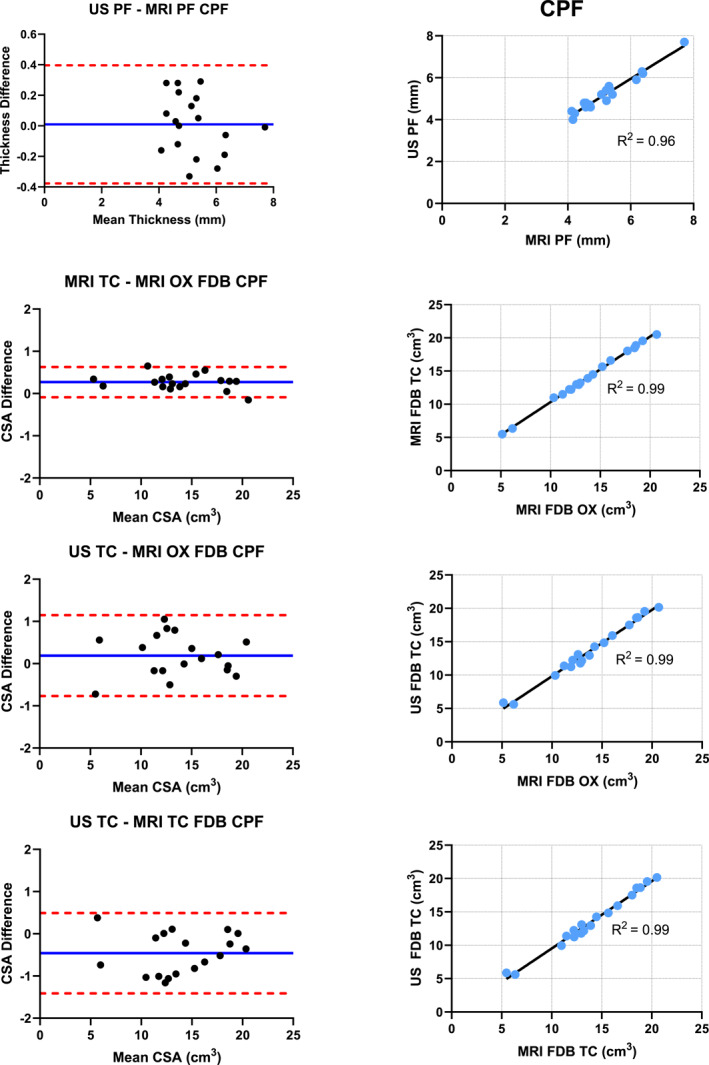

**Figure:**
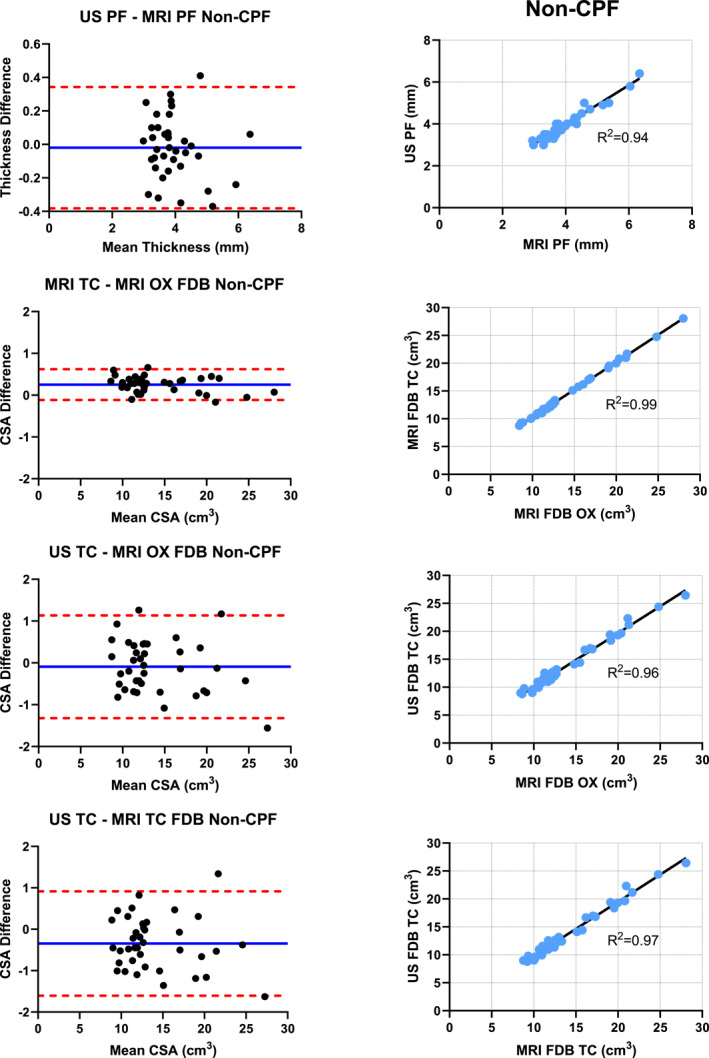

## 3 Results

US and MRI demonstrated excellent repeatability for the plantar fascia and FDB muscle in both study groups. ICCs ranged between 0.989–0.997 (Table 1) for all measures. SEm values ranged from 0.074–0.089 mm for the plantar fascia and between 0.256–0.495 cm^3^ for the FDB muscle across both groups. Relative minimum detectable difference (MDD) values ranged from 3.98%–5.5% for the plantar fascia and 5.06%–9.84% for the FDB muscle across both groups. US measures of the plantar fascia showed comparable SEm and MDD values when compared with MRI indicating similarity of these imaging modalities. However, US measures of the FDB muscle volume had slightly higher SEm and MDD values when compared with MRI in both the non‐PF group and the PF group indicating that US assessment of muscle volume is slightly less sensitive to change over time than either MRI measurement methods (Table 1).

**TABLE 1:** Mean ICC, SEm, absolute and relative MDD values for US and MRI muscle volume (cm^3^), and thickness of plantar fascia (mm).

		ICC (3,1) (95% CI)	SEm (mm) (95% CI)	Absolute MDD (mm)	Relative MDD%
Non‐PF	US—Plantar fascia	0.989 (0.965–0.996)	0.078 (4.073–3.767)	0.216	5.50
	MRI—Plantar fascia	0.990 (0.971–0.996)	0.074 (4.085–3.795)	0.205	5.20
PF	US—Plantar fascia	0.993 (0.950–0.999)	0.075 (5.394–5.046)	0.208	3.98
	MRI—Plantar fascia	0.991 (0.918–0.999)	0.089 (5.384–5.036)	0.247	4.74

		ICC (3,1) (95% CI)	SEm (cm^3^) (95% CI)	Absolute MDD (cm^3^)	Relative MDD%
Non‐PF	FDB MRI—Osirix	0.997 (0.991–0.999)	0.256 (13.538–14.542)	0.710	5.06
	FDB MRI—Truncated cone	0.994 (0.981–0.998)	0.357 (13.590–14.990)	0.990	6.93
	FDB US—Truncated cone	0.988 (0.958–0.997)	0.495 (12.980–14.920)	1.372	9.84
PF	FDB MRI—Osirix	0.994 (0.956–0.999)	0.328 (13.197–14.483)	0.909	6.57
	FDB MRI—Truncated cone	0.991 (0.934–0.999)	0.397 (13.332–14.888)	1.100	7.80
	FDB US—Truncated cone	0.990 (0.950–0.998)	0.425 (12.817–14.483)	1.178	8.63

Very strong correlations were found between US and MRI for the PF and non‐PF groups with values of 0.94–0.96, respectively. FDB muscle correlations between measurement techniques ranged between 0.96 and 0.99 for both the PF and non‐PF groups (Table 2). The high *R* ^2^ value between measurement techniques is one indication of a high level of validity between measurement techniques. These high *R* ^2^ values are seen for both the non‐PF and PF groups. The plantar fascia was significantly thicker in the PF group compared with the non‐PF group (*p* < 0.001). No significant differences were found between the FDB muscle within the groups across measurement techniques or between the PF and non‐PF groups comparing the same measurement technique (*p* > 0.05).

**TABLE 2:** Correlation values and Bland–Altman upper and lower limits of agreement and percent average.

Measurement techniques	*R* ^2^	LoA lower	LoA upper	Bias	LoA % Average
Nonchronic plantar fasciitis					
US—MRI plantar fascia	0.94	−0.38	0.34	−0.02	9.2%
FDB MRI truncated cone—MRI Osirix	0.99	−0.12	0.62	0.25	2.6%
FDB US truncated cone—MRI Osirix	0.96	−1.32	1.13	−0.09	8.8%
FDB US truncated cone—MRI truncated cone	0.97	−1.61	0.92	−0.35	9.0%
Chronic plantar fasciitis					
US—MRI plantar fascia	0.96	−0.38	0.40	−0.01	7.5%
FDB MRI truncated cone—MRI Osirix	0.99	−0.09	0.63	0.18	2.6%
FDB US truncated cone—MRI Osirix	0.99	−0.77	1.15	0.19	4.9%
FDB US truncated cone—MRI truncated cone	0.99	−1.41	0.49	−0.46	6.8%

*Note:* Bias formula is first listed variable subtract second listed variable.

Bland–Altman plots were created across both the PF and non‐PF groups for the plantar fascia thickness and FDB muscle volume comparing all measurement techniques. Bland–Altman limits of agreement percent for the plantar fascia were 9.2% and 7.5% for the non‐PF and PF groups, respectively. Percent limits of agreement ranged from 2.6% to 9.0% across both groups for the FDB measurement comparisons. The bias, upper and lower limits of agreement, and limit of agreement percent are similar when comparing the non‐PF and PF groups across the same measurement technique. These data indicate high levels of validity between measurement techniques. Importantly, it seems that all measurement techniques for the plantar fascia thickness, and the FDB muscle volume have similar levels of validity for the PF and non‐PF groups based upon the *R* ^2^ values and similarity between Bland–Altman plots.

## 4 Discussion

The objective of this study was to assess the repeatability, reliability, and validity of (1) plantar fascia thickness and (2) of a truncated cone volume formula during measurement of an intrinsic foot muscle, the FDB, and between US and MR imaging in aging individuals with and without PF. We found that both US and MRI provide repeatable and reliable assessments of plantar fascia thickness in individuals with and without PF. We found that measurements of muscle volume of the FDB can be reliably and repeatably determined through either the OsiriX auto volume segmenter or by applying a truncated cone volume formula to multiple muscle CSAs. Additionally, we found high validity between measurement techniques across both the PF and non‐PF groups when comparing all measurement techniques for plantar fascia thickness and FDB muscle volume.

The data of this study indicate that US based thickness measures of the plantar fascia are valid when compared with MRI in individuals with and without PF. MRI continues to be regarded as the most sensitive imaging modality when diagnosing plantar fasciitis [47]. However, US use continues to gain clinical popularity in assessing plantar fascia tissue thickness and health and has been listed as a first‐ or second‐line modality for PF diagnosis [48]. Plantar fascia thickness of at least 4 mm is accepted as clinically meaningful in PF diagnosis [49]. Pearson product correlations obtained in this study were very strong (0.94–0.96) for individuals with and without PF suggesting US produced similar plantar fascia thickness measures as MRI. Biases obtained in Bland–Altman plot analysis comparing plantar fascia thickness measured via US to MRI were not statistically significantly different from 0. Additionally, relatively low limit of agreement percents in both groups were found. These data collectively indicates high validity of US based measures of plantar fascia thickness compared with MRI. This information is vital as clinical plantar fascia assessment via US continues to grow. Clinicians and researchers should place high confidence in US based measures of plantar fascia thickness.

FDB muscle volume estimates produced using US truncated cone measurements exhibited very strong Pearson product correlations compared with MRI truncated cone and MRI OsiriX. Correlations seen in this study agree with previously reported single CSA measurements of intrinsic and extrinsic foot muscles [30, 50, 51]. Previously reported Pearson product correlations for single CSA muscle measurements of the FDB were 0.989 [30] and 0.98 [30, 51]. Pearson product correlation values in this study are similar ranging from 0.96 to 0.99 depending on the measurement technique. The high correlation values seen in this study are one indication of a high level of validity between measurement techniques. Further evaluation of validity should include a Bland–Altman plots.

Bland–Altman plot analysis of the measurement techniques of the FDB muscle indicate excellent agreement between US truncated cone—MRI Osirix and very good agreement between US truncated cone—MRI truncated cone and MRI truncated cone—MRI OsiriX. A previous report of limit of Agreement percent between US and MRI in younger healthy individuals for the FDB muscle using a single CSA measurement were 5.7% and 6.6% (Swanson) [30]. This study adds volume estimates of the FDB muscle across various measurement techniques in a pathologic and nonpathologic older population. Our limit of agreement percent ranges from 2.6% to 9.0% across both PF and non‐PF groups. These limit of agreement percent ranges indicate high agreement between measurement techniques in both study groups. The US truncated cone—MRI OsiriX bias in both the PF and non‐PF groups was not significantly different from zero indicating US truncated cone volume and MRI OsiriX volume estimates are statistically the same. The difference between US and MRI truncated cone (US truncated cone—MRI truncated cone) and the difference between the two MRI methods (MRI truncated cone—MRI OsiriX) were significantly different from zero indicating a slight volume adjustment equal to the bias may be needed to accommodate for over/under estimation of muscle size [52]. Inherent differences in imaging between US and MRI including ability to detect fascial layers and resolution and/or differences in formula calculations may account for the needed observed bias adjustment. After examining the Pearson product correlation values together with Bland–Altman plots the authors conclude that the measurement techniques studied have high levels of agreement and may be used interchangeably after slight volume bias adjustments may be made as indicated.

Our results support previous research indicating high reliability for intrinsic foot muscle size as evidenced by high ICC values. Gong et al. determined that correlations between muscle volume and CSA's at varying places along the foot all yield similar and high ICC's, demonstrating that CSA's can act as a surrogate for volume [53]. Additional previous reports including from our lab [30, 31, 51], demonstrate high ICC's for intrinsic foot muscles such as the FDB. Isolation of intrinsic foot muscle force production is unattainable due to overlapping muscle osteokinematic actions with extrinsic foot muscles. Thus, indirect measures of muscle force production, such as muscle CSA or volume, are often used to assess muscle strength. Demonstration of repeatability in both ultrasound and MRI is vital to have confidence in muscle volume measures using these modalities. This study adds continued evidence that high repeatability is obtainable when measuring the FDB muscle in aging individuals with and without PF.

The data of this study contribute that ultrasound and MRI measures of plantar fascia thickness and FDB muscle volume are highly reliable in individuals with and without PF. High reliability is indicated by relatively low error rates as measured via SEm, MDD, and relative MDD %. Previously, Bisi‐Balogun et al. assessed plantar fascia thickness in healthy individuals along the entire fascia, publishing similar SEm ranges of 0.08–0.15 mm as we found in this current study [54]. Regarding the FDB muscle, Abe et al. found a SEm of 0.57 cm^3^ when calculating volume measurements in young healthy adults. Our SEm values for the FDB muscle range from 0.256–0.495 cm^3^ across measurement methods in both groups. Clinicians and researchers may take confidence when calculating plantar fascia thickness and FDB muscle volume using ultrasound based upon these relatively low SEms. The low absolute and relative MDD are further evidence of high reliability and were similar for both PF and non‐PF groups in this study (Table 3). The MDD values from this study in an older population were similar to those found in younger populations as reported by previous researchers [19, 20].

**TABLE 3:** Mean muscle volume (cm^3^).

Measurement technique and modality	Non‐PF	PF
MRI—Plantar fascia	3.94 ± 0.77	5.21 ± 0.94*
US—Plantar fascia	3.92 ± 0.74	5.22 ± 0.89*
MRI—Osirix	14.04 ± 4.67	13.84 ± 4.24
MR—Truncated cone	14.29 ± 4.61	14.11 ± 4.19
US—Truncated cone	13.95 ± 4.52	13.65 ± 4.25

*Note:* No significant differences were found within plantar fasciitis groups between measurement techniques (*p* > 0.05).

^*^ Significant differences between non‐PF and PF groups (*p* < 0.001).

Previously published MRI‐derived estimation of muscle volume yielded reliable and valid estimates in various muscles including the quadriceps [22], rotator cuff muscles [55], gluteus muscles [56], hamstrings [57], and in tissues such as the liver [58]. Each study reported excellent ICC values and MDD volumes ranging from less than 1%–8.91% of the muscle size. Our MRI‐derived estimation of muscle volume average error of 5.65% was within the range of these previously reported studies. Multiple formulas that derive muscle volume from MRI images have been presented in the literature. Among these, the truncated cone formula and cylinder formula have been commonly used to reduce the number of CSA's needed to accurately estimate muscle volume via MRI. This study successfully utilized both these volume calculation methods in MRI and the truncated cone method in US.

Previous studies found variance in muscle estimation accuracy and validity when using the truncated cone and cylinder or Cavalieri formulas via MRI [22, 58]. For instance, the truncated cone formula with MRI has been applied to various other muscles outside the intrinsic foot with disagreements regarding overestimation and underestimation of muscle volume. One study found no significant difference using the truncated cone formula compared to an assumed cylindrical shape when measuring the tibialis anterior, extensor digitorum longus, and extensor hallucis longus muscles [23]. Another study measuring the quadratus femoris found that the truncated cone method consistently underestimated the volume of the muscle when compared to the cylinder formula [24]. Additionally, another study measuring volume via MRI on the quadriceps muscles found that truncated cone was the least accurate of the four methods they used [22], including the cylinder formula which is used by the OsiriX software. We found that the MRI truncated cone method measured a slightly increased volume of the FDB muscle when compared to the OsiriX semiauto volume segmenter and our US volume estimation. Differences in accuracy between methods may be due to the smaller muscle size of the FDB and different shape including multiple points of insertion.

MRI has been found to be a very accurate modality to measure volume and can be used in clinical settings; however, MRI is typically more expensive, takes longer to schedule and return images, and has more contraindications. Ultrasound has various benefits for clinicians including the cost, time spent imaging, availability and portability of the unit, and its user friendliness. Cine loops (short videos showing contraction and relaxation of a muscle) may also increase the precision of ultrasound further promoting its effectiveness [59]. US may be a faster option that can produce an equally reliable volume estimate allowing for confident medical professional use. Based upon the results of this study, researchers and clinicians can repeatably and reliably assess plantar fascia thickness and FDB muscle volume using a truncated cone volume formula via ultrasound when compared with MRI. One potential issue of clinical muscle volume estimation is time needed to perform multiple slice segmentations. Machine learning and artificial intelligence are being used to decrease time spent measuring and calculating muscle CSA and volume.

Recent studies have observed machine learning automatic segmentation of muscle CSA via MRI [60, 61]. Developing software for ultrasound that could better identify and outline muscle bodies would further decrease time spent imaging multiple slices, thus, allowing for a faster measurement of total muscle volume. This may further increase the clinical applicability of ultrasound in healthcare settings. This study did not assess muscle echotexture in addition to muscle volume. Although MRI has the capacity to assess both muscle volume and quality, the primary aim was to determine the agreement between US and MRI in measuring muscle volume specifically. Echotexture assessment via ultrasound introduces subjectivity and was beyond the scope of our primary aim. Although muscle quality is an important clinical consideration, it is not essential to establishing initial agreement between imaging modalities for volumetric measurement [62].

MDD values found by Swanson et al. measuring the FDB were 4.82% [30] and Smith et al. reported less than 10% for the abductor hallucis muscle [28]. The MDD values we found ranged from 4.6% to 9.2% depending on the modality, similarly less than 10% as reported by Smith et al. Despite the MDD being larger for ultrasound compared to MRI this value was still low indicating that US can be useful for estimating muscle volume. Muscle size limits of agreement for measuring the FDB found by Crofts et al. was 17%, Swanson et al. found an average of 6.2% between both feet [30], and our study found 2.3%–9.7% depending on the modalities compared [51]. These values indicate that US can be used to precisely and accurately measure FDB muscle volume. It is likely that our methods would also be able to estimate volume of other intrinsic foot muscles.

## 5 Limitations

We used one common short axis cine loop of the entire FDB muscle scanning proximal to distal and did not use cine loops for each individual slice measurement utilizing a contract relax protocol in our US imaging. Although one common cine loop showed excellent repeatability, reliability, and validity between measurement methods individual cine loops may be researched in the future to determine if an improvement in these metrics is gained.

A second limitation of this study is the ability of the two imaging methods to distinguish muscle characteristics such as fascial borders. Visualization of muscle fascia via MRI is difficult, whereas US often provides greater resolution of the fascia. Thus, muscle measurement tracings using ultrasound in this study were done just inside fascial borders. MRI muscle tracings encapsulated the entire muscle boundary, likely including the muscle fascia border. This would likely lead to larger estimates of muscle volume via MRI as seen in this study.

## 6 Conclusion

Ultrasound is equally repeatable and reliable when calculating plantar fascia thickness and FDB muscle volume compared to the OsiriX semiauto volume segmenter and MRI truncated cone formulas in older adults with and without PF. US is a valid method when measuring plantar fascia thickness compared with MRI. Additionally, utilizing a truncated cone volume formula in conjunction with US imaging shows high validity compared with MRI images analyzed via a truncated cone formula or OsiriX auto volume segmenter for the FDB muscle in older individuals with and without PF.

## Author Contributions

**Derek A. Swanson:** conceptualization, data curation, formal analysis, writing – original draft, writing – review and editing. **Joshua K. Sponbeck:** conceptualization, data curation, formal analysis, writing – original draft, writing – review and editing. **Dallin C. Swanson:** data curation, writing – original draft, writing – review and editing. **Steven P. Allen:** methodology, resources, validation, visualization. **Aaron Wayne Johnson:** conceptualization, methodology, project administration, supervision, writing – review and editing.

## Ethics Statement

Ethical approval was obtained from the Brigham Young University IRB board (IRB2022‐332).

## Conflicts of Interest

The authors declare no conflicts of interest.

## Data Availability

Data are available from the authors upon request.
